# Is the rest perfusion measurement necessary for the diagnosis of myocardial ischaemia in quantitative cardiac perfusion? A CE-MARC sub-study

**DOI:** 10.1186/1532-429X-16-S1-P178

**Published:** 2014-01-16

**Authors:** John D Biglands, Derek R Magee, Steven Sourbron, Sven Plein, John P Greenwood, Aleksandra Radjenovic

**Affiliations:** 1 Division of Medical Physics, University of Leeds, Leeds, UK; 2 School of Computing, University of Leeds, Leeds, UK; 3 MCRC & LIGHT, University of Leeds, Leeds, UK; 4 Institute of Cardiovascular and Medical Sciences, BHF Glasgow Cardiovascular Centre, University of Glasgow, Leeds, UK

## Background

Diagnostic studies using dynamic contrast enhanced (DCE) MRI perfusion imaging typically evaluate perfusion in terms of the myocardial perfusion reserve (MPR), which is the ratio of stress to rest myocardial blood flow (MBF) measurements. The aim of this study was to establish whether or not, in the case of absolute MBF quantitation, the MPR exhibits a diagnostic advantage over the stress MBF measurements alone.

## Methods

This was a retrospective sub-study using data from the CE-MARC trial (Greenwood et al., Lancet, 2012). The CE-MARC trial collected quantitative X-ray angiography data and Single Photon Computed Tomography (SPECT) imaging data as well as DCE-MRI cardiac perfusion data from 752 randomised patients. This allowed a unique gold-standard assessment for the diagnosis of myocardial ischaemia to be generated for this study; being the consensus diagnosis of anatomical (X-ray angiography) and functional (SPECT) imaging. Fifty patients were selected such that the distribution of risk factors and disease status within the sample was representative of the full CE-MARC cohort. Quantitative myocardial blood flow estimates were obtained using four commonly used perfusion models in order to ascertain whether the results were consistent across analysis methodologies. These models were: Fermi-constrained deconvolution, model independent deconvolution, the uptake model and the one compartment model. The three cardiac slices from the MRI data sets were subdivided into 16 segments according to the American Heart Association (AHA) recommendations for perfusion imaging. Rest and stress MBF estimates were established for each of these segments and the MPR was calculated. Using the minimum perfusion score Receiver Operator Characteristic (ROC) curves were then generated using MPR and stress MBF as the diagnostic measure. A DeLong, DeLong, Clarke-Pearson comparison was used to test for statistically significant differences in the Area Under the Curve (AUC) values between the MPR and stress MBF ROC curves.

## Results

**Figure 1:**
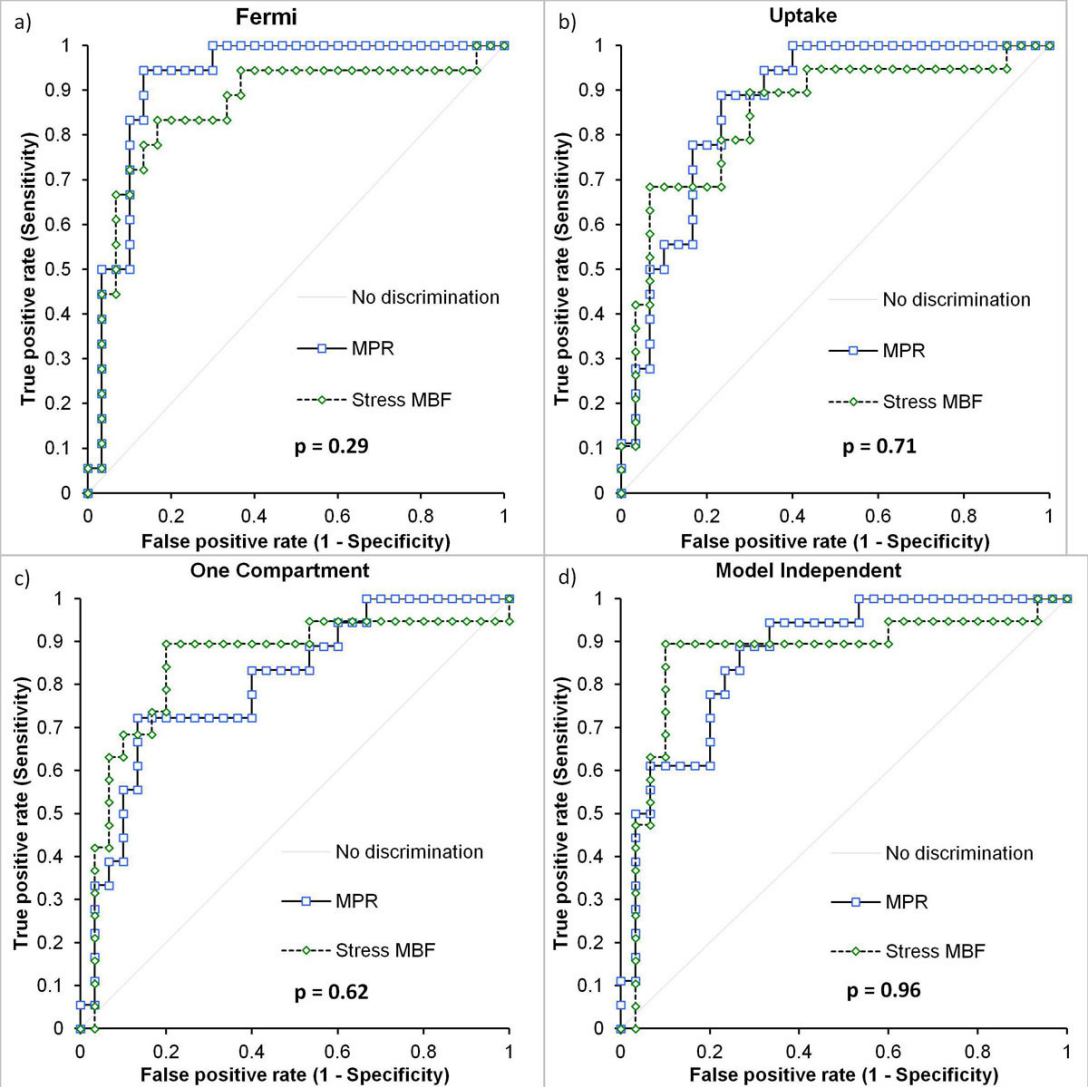
**ROC curves using stress MBF and MPR as the diagnostic measure for a) Fermi-constrained deconvolution, b) the uptake model, c) the one compartment model and d) model independent deconvolution**. DeLong, DeLong Clarke-Pearson p-values for the comparison of the AUC values are shown under the legends.

There was no significant difference in diagnostic performance between stress MBF and MPR with any of the four models (Figure 1). The area under the curve (AUC) values for MPR and stress MBF were: Fermi (0.92, 0.86), Uptake (0.87, 0.85), One compartment (0.80, 0.85) and model independent (0.87, 0.87) respectively.

## Conclusions

Our results demonstrate that stress MBF measurements perform as well as MPR in diagnosing myocardial ischaemia. This implies that the rest MBF measurement does not add any significant information to the diagnosis and could potentially be removed from the investigation protocol without any reduction in diagnostic performance.

## Funding

This work was funded by an NIHR doctoral training fellowship.

